# The molecular signal for the adaptation to cold temperature during early life on Earth

**DOI:** 10.1098/rsbl.2013.0608

**Published:** 2013-10-23

**Authors:** Mathieu Groussin, Bastien Boussau, Sandrine Charles, Samuel Blanquart, Manolo Gouy

**Affiliations:** 1 Laboratoire de Biométrie et Biologie Evolutive, Université de Lyon, Université Lyon 1, CNRS, UMR5558, Villeurbanne, France; 2 Department of Integrative Biology, University of California, Berkeley, CA, USA; 3 Inria Lille Nord Europe, LIFL UMR 8022 (CNRS Université de Lille 1), Villeneuve d'Ascq, France

**Keywords:** non-homogeneous substitution model, ancestral sequence reconstruction, optimal growth temperature, last universal common ancestor, early Earth

## Abstract

Several lines of evidence such as the basal location of thermophilic lineages in large-scale phylogenetic trees and the ancestral sequence reconstruction of single enzymes or large protein concatenations support the conclusion that the ancestors of the bacterial and archaeal domains were thermophilic organisms which were adapted to hot environments during the early stages of the Earth. A parsimonious reasoning would therefore suggest that the last universal common ancestor (LUCA) was also thermophilic. Various authors have used branch-wise non-homogeneous evolutionary models that better capture the variation of molecular compositions among lineages to accurately reconstruct the ancestral G + C contents of ribosomal RNAs and the ancestral amino acid composition of highly conserved proteins. They confirmed the thermophilic nature of the ancestors of Bacteria and Archaea but concluded that LUCA, their last common ancestor, was a mesophilic organism having a moderate optimal growth temperature. In this letter, we investigate the unknown nature of the phylogenetic signal that informs ancestral sequence reconstruction to support this non-parsimonious scenario. We find that rate variation across sites of molecular sequences provides information at different time scales by recording the oldest adaptation to temperature in slow-evolving regions and subsequent adaptations in fast-evolving ones.

## 1. Introduction

Several lines of evidence support the hypothesis that, during early stages of the evolution, life was adapted to high temperatures that may have prevailed on the surface of the early Earth. For instance, previous studies discovered that the deepest branching lineages within the bacterial and archaeal domains are thermophilic [1]. This scenario is also supported by the reconstruction and synthesis of ancestral translation elongation factor Tu sequences that appear more and more thermostable when going back in time [2] and by an estimation of the amino acid composition of ancestral proteomes that appear more similar to the composition of extant thermophiles than that of mesophiles [3].

A tight relation exists between either the G + C content in ribosomal RNAs or the amino acid contents in proteins and the optimal growth temperature (OGT) of Bacteria and Archaea. Such correlations between molecular composition and temperature may be explained by structural adaptation increasing RNA and protein thermostability [4,5] and are likely to remain constant over evolutionary time. They allow the construction of molecular thermometers [6] that can provide estimates of ancestral environmental temperatures if one obtains ancestral base and amino acid compositions through ancestral sequence reconstruction. Using such an approach, Boussau *et al*. [7] concluded that molecular sequence data confirm the hypothesis of high-temperature adaptation during the early stages of life, namely for the ancestors of the bacterial and archaeal domains. However, these authors reported strong evidence for a non-parsimonious scenario in which the last universal common ancestor (LUCA) itself, living at a still earlier stage of the history of life, was a mesophilic organism.

Through a number of control experiments, Boussau *et al*. [7] have shown that the use of non-homogeneous substitution models, which are capable of capturing the variation of composition among lineages, are key to accurately estimate ancestral base and amino acid compositions, and therefore ancestral temperatures. But these authors have not identified the specific molecular properties present in extant sequences that inform non-homogeneous models to support such a non-parsimonious scenario. In this letter, we aim to address this issue.

## 2. Material and methods

### (a) Datasets and non-homogeneous models

Boussau *et al*. [7] built a concatenate of small- and large-subunit rRNAs from 456 organisms (2239 sites) and used the sites restricted to stem regions (1043 sites) to infer the ancestral G + C contents over the tree of life. From these alignments, we selected 125 species covering a broad taxonomic diversity without redundancy in the taxonomic sampling. Regarding the concatenation of proteins, the 56 gene families and 30 species considered in Boussau *et al*. [7] were used here, and increased to 38 species, with the addition of Archaea species in particular, which were poorly represented in the first set of species. We reconstructed ML phylogenetic trees for rRNAs (on the 2239 sites dataset) and proteins with PhyML [8]. A three-domain tree was obtained and the root was placed on the branch between the ancestors of Bacteria and Archaea/Eukaryotes. As in [9] and [7], the branch-wise equilibrium frequencies were estimated along these universal phylogenetic trees. The stem dataset was analysed with the BppML program [10] assuming a discrete gamma distribution with eight categories to model rate variation among sites and the non-homogeneous Galtier & Gouy (GG) substitution model [11]. The GG model specifies branch-wise equilibrium G + C contents, as well as an independent G + C content at the root. For proteins, we used a new branch-wise non-homogeneous model implemented in the maximum-likelihood (ML) framework, named COaLA [12] that we recently designed. See the electronic supplementary material for a description of the COaLA model and an evaluation of the fit to data of the non-homogeneous models in comparison with homogeneous models.

### (b) Molecular thermometers

OGT highly correlates with the G + C content of the stem regions of rRNAs (*ρ* = 0.76, *p*-value < 0.001; see the electronic supplementary material, figure S2) and with the second axis of the COA computed on amino acid compositions of the protein dataset restricted to prokaryotic species (*ρ* = 0.88, *p*-value < 0.001; see the electronic supplementary material, figure S3). We controlled for phylogenetic inertia with the phylogenetic independent contrast approach [13] using the R package APE [14] and observed that those correlations were still strongly significant. Linear regressions between OGTs and compositions were then computed to obtain the molecular thermometers.

### (c) Inference of ancestral compositions and optimal growth temperatures

The ancestral sequences were inferred with BppAncestor [10] using the evolutionary parameters estimated by BppML. For each node of the tree, 100 ancestral sequences were generated by drawing amino acids from the posterior distributions of probabilities. The average composition of these ancestral sequences was calculated and the corresponding ancestral temperatures were deduced from the molecular thermometers (see the electronic supplementary material for the confidence intervals computation and the caution required when interpreting ancestral temperatures).

## 3. Results and discussion

**Figure 1.**
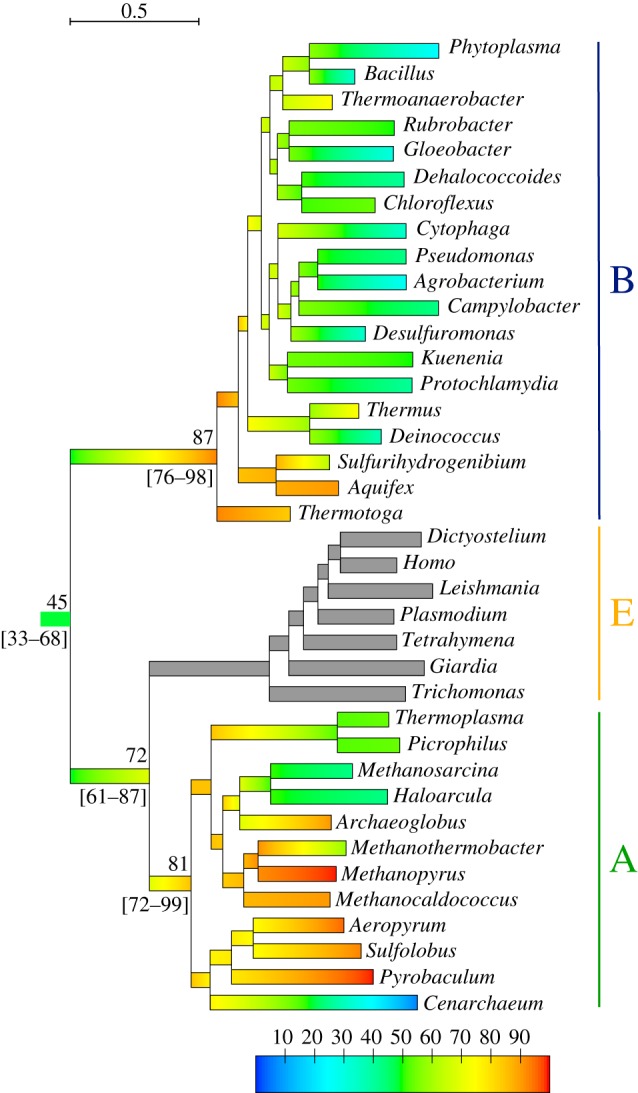
Evolution of OGT along the universal tree of life obtained with the protein dataset. Branches have been coloured according to temperature estimates at nodes, following a linear interpolation from node to node. OGTs for Eukaryotes are not available, their branches are therefore grey coloured. The branch length scale is in substitution/site. The colour scale is in °C. Mean estimates of temperature for LUCA and the ancestors of major domains are given above branches. Confidence intervals (95%) for estimates of ancestral OGTs are given between square brackets.

**Figure 2.**
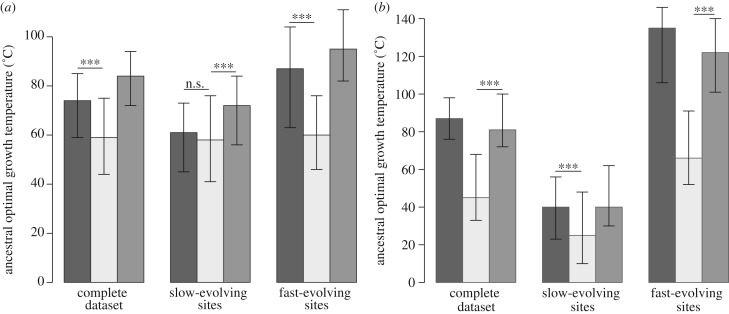
The non-homogeneous models recover the signal for a parallel adaptation to high temperatures within the across-site rate variation. (*a*) rRNA dataset. (*b*) Protein dataset. Ancestral temperatures for domain ancestors and for LUCA were estimated from ancestral compositions inferred with non-homogeneous models, either on all sites of the datasets (complete dataset) or on slow-evolving or fast-evolving sites only. ***p-value < 0.001. n.s. non-significant. Black bars, Bacteria; light grey bars, LUCA; dark grey bars, Archaea.

We first confirm results obtained in [7] with the present rRNA and protein datasets and the non-homogeneous GG [11] and COaLA [12] substitution models in ML. Figures 1 and 2 show that LUCA is estimated to have lived in colder environments than the ancestors of Bacteria and Archaea (Wilcoxon test, *p*-value < 0.001), which were hyperthermophiles. Electronic supplementary material, figure S1 shows that this pattern is also recovered when an alternative tree topology is used, in which Eukaryotes branch within Archaea (Eocyte hypothesis [15]) but is less pronounced with the homogeneous LG model, which infers a thermophilic LUCA.

The phylogenetic signal that informs a non-hyperthermophilic LUCA and yet two hyperthermophilic descendants is currently unknown. However, several points suggest that the variation in evolutionary rate among sites plays a role. First, Fournier & Gogarten [16] highlighted that amino acids that are found in higher proportions in hyperthermophilic species are rarer at slow-evolving sites. Such amino acids notably include charged residues [6]. Second, the signal for a parallel adaptation to high temperatures is partially lost when COaLA is employed without a gamma distribution to model the variation in rate among sites (see the electronic supplementary material, figure S1).

To highlight the influence of rate variation among sites in the differential recording of ancestral compositions, we partitioned the rRNA and protein datasets according to the site evolutionary rates. Figure 2*a*,*b* shows that with slow-evolving sites, all ancestors are inferred to be mesophilic organisms, LUCA being adapted to lower temperatures than its two descendants. The ancestral compositions of fast-evolving sites tend to favour hotter ancestral environments, even for LUCA with proteins. But LUCA is still inferred to live at lower temperatures than the ancestors of Bacteria and Archaea. As expected, the quantitative estimates of past temperatures inferred by both slow- and fast-evolving sites are different from those obtained with the complete dataset. Indeed, although slow-evolving sites conserved reliable signals for ancestral compositions, they carry less phylogenetic information for the early parallel adaptation to high temperature, which explains why this pattern is less pronounced than that with the complete dataset. However, both the G + C content (rRNAs) and the third axis of a correspondence analysis (proteins) computed from the slow-evolving sites of extant sequences correlate with OGT (*ρ* = 0.72, *p*-value < 0.001 and *ρ* = 0.43, *p*-value < 0.05, respectively), adding support to the idea that slow-evolving sites can respond to temperature and can represent accurate fossils of ancestral adaptation to temperature. Fast-evolving sites contain a stronger signal for this parallel adaptation but necessarily less reliable information for ancestral compositions, and so ancestral temperatures.

All these results suggest the presence of a genuine signal in molecular sequences indicating a mesophilic LUCA. This signal was recorded thanks to a combination of compositional variation in time and rate variation in site such that slow-evolving sites more accurately reflect older temperatures, while fast-evolving sites partially erased this oldest signal in favour of subsequent adaptations to higher temperatures.

Gowri-Shankar & Rattray [17] showed that there is an intrinsic correlation between evolutionary rates across sites and base composition in rRNAs. Therefore, nucleotide composition varies across the sites of an rRNA alignment. These authors showed that branch-wise non-homogeneous models, which account for the variation of composition in time but assume across-site homogeneity, may infer biased ancestral sequence compositions for sequences generated by a time-homogeneous process in which evolutionary rate and base compositions are correlated. The inference bias is directed towards the composition of slow-evolving sites which are, in the case of full-length rRNAs including both stem and loop regions, GC-poor. One could therefore wonder whether such an inference bias would be responsible for the low G + C content inferred for LUCA compared with the higher G + C contents of its first descendants. We reject this bias with two points. First, as in this study, Boussau *et al*. [7] applied the molecular thermometers on rRNAs to only the stem regions of the molecule. Electronic supplementary material, figure S4 shows that, for these regions, the correlation found by Gowri-Shankar & Rattray [17] is in the opposite direction, although non-significant, with G + C-enriched slow-evolving sites. Second, we simulated data in a context where the bias would apply, assuming only heterogeneity among sites and no heterogeneity among branches, and verified whether the correlation between site evolutionary rates and site compositions incorrectly informs the non-homogeneous model to estimate a lower G + C content of LUCA than for its descendants. We partitioned rRNA alignment sites in eight categories according to their evolutionary rate. For each rate-specific category, we simulated DNA sequences with a homogeneous Tamura92 model and the G + C equilibrium frequency fixed to the observed G + C frequency of the category, and then concatenated the eight simulated sets. We repeated this procedure 100 times and reconstructed ancestral G + C contents with the non-homogeneous GG model on each concatenated simulated alignment. Electronic supplementary material, figure S5 shows that the pattern of parallel increase in G + C content from LUCA found from real data is not recovered. Instead, LUCA has a higher G + C content than its two descendants. As slow-evolving sites of stem regions have globally higher G + C contents than fast-evolving ones (see the electronic supplementary material, figure S4), this simulation result is in agreement with the bias of Gowri-Shankar & Rattray [17]. It further suggests that, if the non-homogeneous model applied to real data is affected by the bias as it is when applied to simulations, the true G + C content of LUCA may so far have been overestimated.

All these results indicate that non-homogeneous models can capture a genuine timewise variation in composition and that the pattern of parallel increase to high temperatures does not result from a bias owing to a correlation between site-specific rates and site-specific compositions [17] but emerges in spite of this bias.

## Data accessibility

Data are available at dryad digital repository (http://datadryad.org/): doi:10.5061/dryad.90525.
